# Endometrial Histopathology in Abnormal Uterine Bleeding and Its Relation With Thyroid Profile and Endometrial Thickness

**DOI:** 10.7759/cureus.37931

**Published:** 2023-04-21

**Authors:** Harshita Deep Sahu, Amit V Varma, Srushti Karmarkar, Kamal Malukani, Anushka Khanuja, Pooja Kesharwani

**Affiliations:** 1 Pathology, Sri Aurobindo Medical College and Post-graduate Institute, Indore, IND

**Keywords:** postmenopausal women, peri-menopausal, endometrial histopathology, dysfunctional uterine bleeding, thyroid function test, endometrial thickness, abnormal uterine bleeding

## Abstract

Introduction: Abnormal uterine bleeding (AUB) is a common complaint in postmenopausal and perimenopausal women, caused by a range of disorders, including structural and systemic diseases. The evaluation of endometrial thickness (ET) via radiological methods, followed by a histopathological examination of the endometrium, is useful for proper diagnosis. Among systemic diseases, thyroid dysfunction, specifically hypothyroidism and hyperthyroidism, contribute significantly to AUB cases.

Materials and methods: This descriptive cross-sectional study was conducted at Sri Aurobindo Medical College, Indore, Madhya Pradesh, India, over a period of 16 months, from May 2021 to September 2022. Patients presenting with abnormal uterine bleeding and undergoing thyroid function tests (TFTs), ultrasonography, and endometrial biopsy/hysterectomy at the gynecological outpatient department were included. Hospital records were used to obtain clinical details and investigation results. Endometrial thickness and thyroid status were recorded, and descriptive statistics were used to analyze the collected data.

Results: This study included 150 patients with abnormal uterine bleeding, with a mean age of 44 years and 80.6% of patients in the premenopausal age group. A total of 48% of patients had a deranged thyroid profile, with hypothyroidism being more common (91.6%). Structural causes of AUB were identified in 81.3% of cases, with adenomyosis (33.65%), concomitant adenomyosis and leiomyoma (31.5%), and leiomyoma (14.8%) being the most common. Endometrial polyps (4.6%) and endometrial carcinoma (0.6%) were also observed and were consistent with the final histopathology. The remaining 18 patients had no structural causes and were categorized as cases of dysfunctional uterine bleeding (DUB). Increased ET was more commonly observed in postmenopausal patients (4.3%) compared to premenopausal patients (0.7%) among those with AUB, while the reverse was true for patients with DUB. Increased ET was commonly associated with hypothyroidism in both groups. Histopathological examination of endometrial biopsies/hysterectomy specimens revealed additional findings in some patients, including hyperplasia of the endometrium with (0.7%) and without atypia (4%), leading to a more accurate diagnosis.

Conclusion: AUB is a prevalent condition affecting women in both pre-menopausal and postmenopausal stages, frequently caused by structural anomalies. However, thyroid dysfunction, especially hypothyroidism, is also a significant contributing factor. As such, thyroid function tests (TFTs) are an effective and economical means of identifying potential underlying causes of AUB. Hypothyroidism is frequently associated with increased endometrial thickness, and histopathological examination remains the gold standard for determining the precise cause of AUB.

## Introduction

Abnormal uterine bleeding (AUB) refers to variations in the duration, frequency, and volume of blood loss during menstruation [1]. It affects approximately 10-20% of women aged 15-50 and about 50% of perimenopausal women [2]. AUB encompasses a diverse range of disorders, including reproductive tract diseases, systemic diseases, and iatrogenic causes. Clinically, it can present in numerous forms, such as menorrhagia, metrorrhagia, menometrorrhagia, polymenorrhea, polymenorrhagia, and oligomenorrhea [3]. Thyroid dysfunction, including both hypothyroidism and hyperthyroidism, accounts for 30%-40% of systemic disorders causing AUB and can be readily diagnosed by thyroid function tests (TFTs) [4].

Transvaginal ultrasonography (USG) is an invaluable tool for measuring endometrial thickness (ET) and patterns, and identifying organic causes such as leiomyomas and endometrial malignancies [5]. An ET threshold of ≥4 mm in postmenopausal women and ≥14 mm in premenopausal women indicates an underlying endometrial pathology and warrants histopathological evaluation of the endometrium [6]. Histopathology of endometrial sampling remains the gold standard for evaluating endometrial neoplasia, hyperplasia, metaplasia, and pill-induced and functional endometrial abnormalities [3]. The present study aims to explore the relationship between the thyroid profile, ultrasonographical endometrial thickness, and histopathological findings of the endometrium in cases of AUB.

## Materials and methods

This study is a descriptive cross-sectional investigation that was conducted at the Department of Pathology, Sri Aurobindo Medical College, Indore, Madhya Pradesh, India, over a period of 16 months, from May 2021 to September 2022. Sri Aurobindo Institute of Medical Sciences (SAIMS) institutional review board (IRB) and institutional ethics committee (IEC) approval was obtained (IEC number: SAIMS/RC/IEC/2021/225). The study included patients who visited the gynecological outpatient department with complaints of abnormal uterine bleeding and underwent thyroid function tests, ultrasonography, and endometrial biopsy/total abdominal hysterectomy (TAH) or vaginal hysterectomy.

Women who reported menstrual irregularities such as heavy menstrual bleeding, intermenstrual bleeding, and breakthrough bleeding were enrolled in the study. Medical records were reviewed to obtain a comprehensive medical history, TFT results, and USG reports. TFT was done using a chemiluminescence immunoassay (CLIA), which is a highly sensitive method. Normal ranges for T3, T4, and thyroid stimulating hormone (TSH) by this method were 0.8 to 2.0 ng/ml, 5.1 to 14.1 ng/ml, and 0.27 to 4.2 mIU/ml. The study excluded cases of AUB resulting from gestational causes as well as pregnant patients and those with bleeding disorders. The study also excluded cases of bleeding due to iatrogenic causes such as hormone and steroid intake in the preceding three months, as well as those due to the use of contraceptive devices. Endometrial samples obtained from biopsy, curettage, and hysterectomy specimens in the Department of Pathology were analyzed. A standard protocol was used for grossing both endometrial biopsy and hysterectomy specimens. The study used descriptive statistics to analyze the data collected.

## Results

The study included a total of 150 cases of AUB, all of whom underwent TFT and endometrial biopsy or TAH or vaginal hysterectomy. Out of these cases, 32 patients underwent endometrial biopsy, while 118 patients underwent either TAH or vaginal hysterectomy. The age of patients ranged from 28 to 79 years, with a mean age of 44 years. The distribution of patients according to the age group is presented in Table 1*.*

**Table 1:** Age group versus the number of patients.

Age (years)	No. of cases	Percentage of cases (%)
<30	02	1.3
31-40	41	27.3
41-50	79	52.6
51-60	23	15.3
>60	05	3.3
Total	150	100

The bulk of patients (121; 80.6%) were classified as premenopausal, while merely 29 (19.4%) were postmenopausal. In terms of symptom duration, most patients (74; 49.3%) reported abnormal bleeding for one to three months, 64 (42.6%) reported it for four to six months, and only 3 (2%) reported it for over 12 months. Dysmenorrhea was the most common complaint observed in this study, affecting 64 (42.6%) patients, followed by menorrhagia in 5 (34%) patients. Less common patterns were observed, with 16.5% of patients experiencing polymenorrhea and 7.4% experiencing intermittent bleeding. Table* *2 presents the distribution of patients according to bleeding pattern and thyroid dysfunction.

**Table 2:** Bleeding pattern and thyroid dysfunction versus the number of patients.

Bleeding pattern/complaint	No. of patients (n) (%)	Euthyroid	Hypothyroid	Hyperthyroid
Dysmenorrhoea	64 (42.6)	32	29	03
Menorrhagia	52 (34)	26	24	02
Polymenorrhea	24 (16.5)	14	10	00
Intermittent bleeding	10 (7.4)	06	03	01
Total	150	78	66	06

All patients had their TFT results available. Out of the total AUB cases, 72 (48%) were found to have thyroid dysfunction in the form of either hyperthyroidism or hypothyroidism, while the remaining 78 (52%) were euthyroid. Hypothyroidism was the predominant condition and was observed in 66/72 (91.6%) patients, whereas hyperthyroidism was seen in only 6/72 (8.3%) patients. Table 3* *shows the distribution of patients according to thyroid dysfunction.

**Table 3:** Thyroid dysfunction versus the number of patients.

Thyroid status	No. of cases	Percentage of cases (%)
Hypothyroid	66	44
Hyperthyroid	06	04
Euthyroid	78	52
Total	150	100

Thyroid dysfunction was predominantly observed in the age groups of 41-50 years and 31-40 years, with hypothyroidism being the most prevalent. In the former age group, 32/78 patients (41.5%) were hypothyroid, while only 3.8% of patients were hyperthyroid. In the latter age group, 21/42 (50%) were hypothyroid and only 4.7% were hyperthyroid. Among patients aged 51-60 years, 12/23 (52.1%) were hypothyroid and only one patient was hyperthyroid. Table 4* *presents the distribution of thyroid dysfunction across different age groups. 

**Table 4:** Thyroid dysfunction in different age groups.

Age (years)	No. of cases (n) (%)	Euthyroid (n) (%)	Hypothyroid (n) (%)	Hyperthyroid (n) (%)
<30	03 (2)	03 (4)	00	00
31-40	42 (28)	19 (24)	21 (32)	2 (33)
41-50	78 (52)	43 (55)	32 (49)	3 (50)
51-60	23 (15)	10 (13)	12 (18)	1 (16)
>60	04 (3)	03 (4)	1 (1.5)	00
Total	150	78	66	06

Out of the 150 patients, 55 (33.6%) were diagnosed with adenomyosis (AUB-A) on clinico-radiological examination. Additionally, 47 (31.5%) patients were diagnosed with concomitant adenomyosis and leiomyoma (AUB-A+L), 22 (14.8%) cases were diagnosed with only leiomyoma (AUB-L), seven cases were diagnosed with endometrial polyps (AUB-P), and a single case was diagnosed as carcinoma endometrium (AUB-M). In all of these cases, the clinico-radiological diagnosis was in agreement with the final histopathological diagnosis. The remaining 18 cases did not have any identifiable structural cause of AUB on gross or histopathological examination of the uterus. These cases were classified as dysfunctional uterine bleeding (DUB). Table 5 provides a distribution of patients based on the clinico-radiological diagnosis.

**Table 5:** Clinico-radiological diagnosis versus the number of patients. AUB: abnormal uterine bleeding.

Structural causes of AUB	No. of cases	Percentage (%) of cases
Adenomyosis	55	36.6
Adenomyosis + leiomyoma	47	31.3
Adenomyosis + leiomyoma + polyp	01	0.6
Adenomyosis + polyp	03	02
Leiomyoma	22	15
Leiomyoma + polyp	01	0.6
Malignancy	01	0.6
Polyp	02	1.3
Dysfunctional uterine bleeding	18	12
Total	150	100

USG was employed to evaluate ET in all 150 patients. A normal ET cut-off of 14 mm and 4 mm was considered for premenopausal and postmenopausal patients, respectively. Among the 106 premenopausal patients with AUB, those with thyroid dysfunction showed normal ET, except for a single euthyroid patient with an increased ET of 22 mm. Of the 26 postmenopausal patients with AUB, only one hypothyroid patient had an increased ET of 6 mm, while 22 patients had normal ET on USG. Three euthyroid patients exhibited an increased ET of 10 mm, 6 mm, and 5 mm, respectively. Table 6* *depicts the distribution of AUB patients (n=132) with structural causes based on thyroid dysfunction and endometrial thickness.

**Table 6:** Thyroid dysfunction and ET versus the number of patients of AUB with structural causes. AUB: abnormal uterine bleeding, ET: endometrial thickness.

	Premenopausal (n=106)		Postmenopausal (n=26)	
Thyroid status	Normal ET (n) (%)	Increased ET (n) (%)	Normal ET (n) (%)	Increased ET (n) (%)
Euthyroid	53 (50)	01 (0.9)	09 (35)	01 (0.9)
Hypothyroid	47 (44)	00	13 (50)	03 (8)
Hyperthyroid	05 (5)	00	00	00
Total (n=132)	105 (79.5)	01 (0.7)	22 (16.6)	04 (3)

In the case of DUB, 16 premenopausal patients were evaluated, out of whom three patients with hypothyroidism and one euthyroid patient had an increased ET of 16 mm, 18 mm, 19 mm, and 15 mm, respectively. Two postmenopausal patients with DUB were also assessed, one of whom had hypothyroidism and an increased ET of 19 mm. Table 7 depicts the distribution of DUB patients (n=18) based on thyroid dysfunction and endometrial thickness. 

**Table 7:** Thyroid dysfunction and ET versus the number of patients of DUB. ET: endometrial thickness, DUB: dysfunctional uterine bleeding.

	Premenopausal (n=16)		Postmenopausal (n=02)	
Thyroid status	Normal ET (n) (%)	Increased ET (n) (%)	Normal ET (n) (%)	Increased ET (n) (%)
Euthyroid	11 (68)	01 (7)	01 (50)	00
Hypothyroid	01 (7)	03(19)	00	01 (50)
Hyperthyroid	00	00	00	00
Total (n=18)	12 (66.6)	04 (22.2)	01 (5.5)	01 (5.5)

On histopathological examination, the endometrial patterns observed in 139/150 patients were indicative of a normal cyclical pattern of proliferative and secretory endometrium, accounting for 92.6% of the total patient population. Among these patients, a predominantly proliferative pattern was detected in 119 (79.3%) cases, while a secretory pattern was found in 20 (13.3%) patients. In 6 (4%) cases, hyperplasia of the endometrium without atypia was identified. One patient had hyperplasia with atypia, and three cases showed the presence of an endometrial polyp. Only one patient (0.7%) was diagnosed with a malignant lesion, specifically endometrioid carcinoma of the endometrium. Table 8 presents the distribution of patients with thyroid dysfunction and their corresponding endometrial pathology.

**Table 8:** Endometrial histopathology and thyroid dysfunction versus the number of patients.

Endometrial histopathology	Proliferative endometrium	Secretory endometrium	Hyperplastic endometrium	Hyperplastic endometrium with atypia	Carcinoma	Polyp
Euthyroid (n=78)	69	06	01	00	01	01
Hypothyroid (n=67)	47	12	05	01	00	02
Hyperthyroid (n=5)	03	02	00	00	00	00
Total (n=150)	119	20	06	01	01	03
Percentage (%)	79.3	13.3	4	0.7	0.7	2

Of the total cases, the majority were euthyroid (52%), followed by hypothyroid (45%), and hyperthyroid (4%). Among the euthyroid patients, a maximum of 88% had a proliferative phase endometrium, while 8% had a secretory phase endometrium. Hyperplastic endometrium without atypia and carcinoma of the endometrium were observed in a single case each. Among the hypothyroid patients, 70.2% had a proliferative phase endometrium, while 18% had a secretory phase endometrium. Hyperplastic endometrium without atypia, hyperplastic endometrium with atypia, and endometrial polyps were found in 7.5%, 1.4%, and 3% of patients, respectively. Patients with hyperthyroidism had either proliferative (3/5) or secretory phase endometrium (2/5).

## Discussion

Abnormal uterine bleeding (AUB) is a frequently encountered condition among females, particularly in the perimenopausal age group, which spans the fourth to fifth decades of life [7]. In the present study, the majority of AUB patients (52.6%) belonged to the age group of 41-50 years, with 27.3% in the 31-40 years age bracket. In previous studies conducted by Lohith et al. [8] and Thakur et al. [2], 41.7% and 24% of their patients, respectively, were aged 41-50 years, and a significant percentage (43% and 35.3%, respectively) were in the younger age group of 20-30 years. Our study showed a higher mean age of patients (44 years) compared to previous studies done by Thakur et al. [2] and Jaiswal et al [9]. However, the mean age in our study was comparable to that observed by Lohith et al [8].

The majority (80.6%) of patients in our study were premenopausal, while 19.4% were postmenopausal, consistent with findings from other studies by Kattel et al. [10], Koirala et al. [11], and Jaiswal et al [9]. However, Lohith et al. [8] observed that 81.8% of their patients to be in the perimenopausal age group. Regarding the presenting complaints or patterns of bleeding observed in our study, dysmenorrhea was reported by 42.6% of patients, followed by menorrhagia in 34% of patients. Sebtain et al. [7] and Kattel et al. [10] in their studies found menorrhagia to be the most common pattern of bleeding, observed in 47.6 % and 36.7 % of patients, respectively.

Thyroid dysfunction was observed in 48% (72/150) of the patients in our study, with a higher prevalence seen in the age group of 31-50 years. Hypothyroidism was the more common form of thyroid dysfunction, affecting 45% (66/150) of the patients, whereas only 4% (6/150) had hyperthyroidism. The remaining 52% of the patients were euthyroid. Similar findings were reported by Sebtain et al. [7] in a study comprising 500 patients, with hypothyroidism observed in about 30% of the patients. In another study by Koirala et al. [11], hypothyroidism was also more common (27%) in a similar age group, with only 8% of the patients having hyperthyroidism.

Among the various structural causes of AUB diagnosed in our study, adenomyosis was the most common, affecting 33.6% of the patients, followed by concomitant adenomyosis and leiomyoma in 31.5% of the patients. In contrast to our findings, Lohith et al. reported leiomyoma as the most common structural cause of AUB (60.6%) in a study comprising of 132 patients, followed by concomitant adenomyosis and leiomyoma (14.4%) [8]. Kattel et al. also observed leiomyoma as the most common cause (26.7%), followed by adenomyosis (5.5%) [10]. Other less common causes in our study included endometrial polyp and endometrial carcinoma, seen in 1.3% and 0.6% of the cases, respectively.

DUB is a diagnosis that is made after excluding other underlying causes. In our study, 12% (18/160) of the patients were diagnosed with DUB. A majority of the patients in our study (94.6%) had a normal endometrial thickness on ultrasound, which is consistent with previous studies by Khanna et al. [12] and Jaiswal et al. [13], who reported 85% and 78% of patients, respectively, with a normal endometrial thickness.

Increased endometrial thickness was observed in four (15.3%) patients with AUB in our study, comprising two patients with adenomyosis, one patient with adenomyosis and leiomyoma, and one patient with endometrial carcinoma, confirmed by histopathology. Of these patients, three were hypothyroid and one was euthyroid, and all were postmenopausal. Out of the 18 patients with DUB, 5 (27.7%) had increased endometrial thickness. Four premenopausal patients had hyperplastic endometrium without atypia, and one postmenopausal patient had hyperplastic endometrium with atypia, as confirmed by histopathology. Of these patients, four were hypothyroid, and one was euthyroid. Hence, hypothyroidism was found to be more commonly associated with increased endometrial thickness and underlying endometrial pathology.

In our study, the majority (92.6%) of patients showed a normal cyclical pattern of either proliferative or secretory endometrium upon histopathological analysis, which is consistent with previous research [8,14]. Hyperplasia of the endometrium without atypia was observed in 4% of patients, while hyperplasia with atypia was only seen in a single patient. In studies conducted by Lohith et al. [8] and Koirala et al. [11], the occurrence of hyperplasia without atypia was higher, observed in 8.4% and 8.2% of patients, respectively.

Endometrial polyps were diagnosed in three (2%) cases on the basis of histopathology, which is comparable to the findings reported by Bhat et al. (4%; 8/200) [15]. Gorla et al. [16] found endometrial polyps in 6.6% (18/240) of patients. The incidence of malignant lesions was low in our study, with only one patient diagnosed with endometrioid adenocarcinoma of the endometrium upon histopathological examination, which is consistent with previous studies by Khanna et al. (1.2%) [13], Bhat et al. [15], and Chhatrasal et al. (1.8%) [17]. However, Agarwal et al. [18] reported a significantly higher incidence of 4.8% cases of endometrial carcinoma than observed in our study.

In our study, the proliferative pattern was common in both hypothyroid and hyperthyroid patients, observed in 70% and 60% of patients, respectively. Koirala et al. [11] also reported the proliferative pattern as a common pattern in both hypothyroidism and hyperthyroidism, observed in 45% and 36.3%, respectively. The incidence of endometrial hyperplasia in this study was found to be 5%, which is comparable to a study by Lohith et al. [8], reporting an 8.4% incidence. However, Koirala et al. [11], Bhat et al. [15], and Agarwal et al. [18] reported slightly higher incidences of endometrial hyperplasia (8.1%, 9%, and 9.6%, respectively). Iqbal et al. [19] reported a significantly higher incidence of endometrial hyperplasia (14.7%). However, it should be noted that the small sample size of our study may limit the generalizability of our observations.

## Conclusions

In light of our findings, it is evident that AUB affects a broad spectrum of females, including those in both premenopausal and postmenopausal stages, with varied menstrual irregularities being the most common presenting complaints. Structural abnormalities, particularly adenomyosis and leiomyomas, are frequently associated with AUB. Notably, thyroid dysfunction, particularly hypothyroidism, plays a pivotal role in the etiology of AUB. Therefore, it is crucial to evaluate thyroid function tests while investigating a patient with AUB. This readily available, cost-effective, and rapid method can aid in the diagnosis of a potentially treatable cause of AUB, thereby avoiding unnecessary hormonal treatments and invasive procedures like hysterectomies. Furthermore, increased endometrial thickness is more prevalent in hypothyroidism and often indicates an underlying endometrial pathology. Therefore, histopathological examination of the endometrium remains the gold standard for diagnosing the specific cause of AUB.
